# Neutrophil Microvesicles from Healthy Control and Rheumatoid Arthritis Patients Prevent the Inflammatory Activation of Macrophages

**DOI:** 10.1016/j.ebiom.2018.02.003

**Published:** 2018-02-07

**Authors:** Hefin I. Rhys, Francesco Dell'Accio, Costantino Pitzalis, Adrian Moore, Lucy V. Norling, Mauro Perretti

**Affiliations:** a The William Harvey Research Institute, Barts and The London School of Medicine and Dentistry, Queen Mary University of London, Charterhouse Square, London EC1M 6BQ, United Kingdom; b Centre for inflammation and therapeutic innovation, Queen Mary University of London, Charterhouse Square, London EC1M 6BQ, United Kingdom; c UCB Pharma, Bath Road, Slough, United Kingdom

**Keywords:** Neutrophils, Macrophages, Vesicles, Rheumatoid arthritis

## Abstract

Microvesicles (MVs) are emerging as a novel means to enact cell-to-cell communication in inflammation. Here, we aimed to ascertain the ability of neutrophil-derived MVs to modulate target cell behaviour, the focus being the macrophage.

MVs were generated in response to tumour necrosis factor-α, from healthy control neutrophils or those from rheumatoid arthritis patients. MVs were used to stimulate human monocyte-derived macrophages *in vitro*, or administered intra-articularly in the K/BxN mouse model of arthritis. A macrophage/fibroblast-like synoviocyte co-culture system was used to study the effects of vesicles on the crosstalk between these cells.

We demonstrate a direct role for phosphatidylserine and annexin-A1 exposed by the MVs to counteract classical activation of the macrophages, and promote the release of transforming growth factor-β, respectively. Classically-activated macrophages exposed to neutrophil MVs no longer activated fibroblast-like synoviocytes in subsequent co-culture settings. Finally, intra-articular administration of neutrophil MVs from rheumatoid arthritis patients in arthritic mice affected the phenotype of joint macrophages.

Altogether these data, with the identification of specific MV determinants, open new opportunities to modulate on-going inflammation in the synovia – mainly by affecting macrophage polarization and potentially also fibroblast-like synoviocytes - through the delivery of autologous or heterologous MVs produced from neutrophils.

## Highlights

• Neutrophil microvesicles restrict the inflammatory activation of macrophages by presenting phosphatidylserine to Mer.
• Annexin A1 expressed on neutrophil microvesicles induces macrophage release of TGFβ by activating FPR2.
• Neutrophil microvesicles restrict the ability of macrophages to activate fibroblast-like synoviocytes.

All cells release small, spherical parcels of information, called vesicles, that they use to send signals between each other. One immune cell type, the neutrophil, has been shown to release vesicles which prevents other immune cells from becoming hyperactive: herein we focus on rheumatoid arthritis. This paper demonstrates that vesicles released from neutrophils are able to prevent another immune cell type, the macrophage, from becoming activated. The importance of this is that this mechanism is occurring naturally, and if we can understand it, we may be able to use our own bodies' mechanisms to control chronic inflammation.

## 1 Introduction

Released directly from the plasma membrane of virtually all cells in response to calcium signalling, microvesicles (MV; also termed microparticles or more generically extracellular vesicles) are right-side out, double membrane-enclosed structures with a 100–1000 nm diameter. The varied composition of MV, which contain lipids, proteins and nucleic acids, and downstream efficacy depend upon their cellular source and activation stimulus (Van der Pol et al., 2012). Present in a variety of biological fluids, MVs can impart both homeostatic and pathophysiological functions on local and distant tissues.

Despite their propensity to drive acute inflammation, neutrophils are one of few populations of cells whose MVs are known to promote tissue protection, and in some cases repair, by affecting function and phenotype of target cells (Dalli et al., 2008; Eken et al., 2008; Gasser and Schifferli, 2004). Of these, macrophages are central to the recovery of homeostasis after an inflammatory insult, and controlling their phenotype is desirable in chronic inflammatory diseases, including rheumatoid arthritis (RA). Macrophages are remarkably plastic in their ability to change phenotype and respond to a wide spectrum of stimuli. In response to the T_h_1 cytokine interferon gamma (IFN-γ) and toll-like receptor ligands such as lipopolysaccharide (LPS), macrophages upregulate expression of major histocompatibility complex type II (MHCII), the co-stimulatory molecule CD86 and interleukin (IL)-12 and IL-1β (Bosedasgupta and Pieters, 2014; Biswas and Mantovani, 2010). In RA, these pro-inflammatory macrophages drive disease progression and cartilage erosion by i) recruiting other immune cells (Misharin et al., 2014; Vogelpoel et al., 2014), ii) promoting fibroblast-like synoviocyte (FLS) activation (Wilkinson et al., 1993), and iii) undergoing osteoclastogenesis (Fujikawa et al., 1996).

At the polar end of the macrophage phenotype spectrum, macrophages stimulated with T_h_2 cytokines such as IL-4, express scavenger receptors like CD206 and the anti-inflammatory cytokines IL-10 and transforming growth factor-β (TGF-β) (Gordon, 2003). These pro-resolution/wound healing macrophages reduce leukocyte recruitment (Dean et al., 2008; Bellac et al., 2014), exhibit efferocytic capacity to remove apoptotic neutrophils (Zizzo et al., 2012; McCauley et al., 2014) and promote tissue remodelling and repair (Huynh et al., 2002; Rigamonti et al., 2014; Harel-Adar et al., 2011). While these distinct, polar phenotypes are readily generated *in vitro*, whether macrophages in complex tissues separate into discrete populations seems to depend on the tissue/disease context (Martinez and Gordon, 2014). Of note, macrophage heterogeneity in the arthritic synovium has not yet been fully characterised (Ma and Pope, 2005), nonetheless, attenuating or even reversing a pro-inflammatory polarization in synovial macrophages may favour resolution and repair within the inflamed joint.

We have previously demonstrated that neutrophil-derived MVs promote chondrocyte survival and proteoglycan deposition by stimulating TGF-β production (Headland et al., 2015). Such an effect was partly reliant on the pro-resolution protein annexin A1 (anxA1; Perretti et al., 2017). There is some evidence that neutrophil MVs can modulate macrophage responses (Eken et al., 2013; Dalli and Serhan, 2012), however how these effects are attained is only partly understood. At the same time, the downstream consequences of macrophage exposure to neutrophil-derived MVs have not been investigated.

Here we studied the properties of neutrophil MVs on human monocyte-derived macrophages identifying at least some of the effectors responsible for changes in cell polarization. Moreover, we investigate how such changes would impact downstream on macrophage/FLS crosstalk. Of potential therapeutic relevance, the most salient effects were replicated by MVs prepared from RA patient circulating neutrophils.

## 2 Materials and Methods

### 2.1 Patients

All volunteers gave written, informed consent to blood collection and the procedure was approved by the East London & The City Local Research Ethics Committee references 05/Q0603/34 and 07/Q0605/29 for healthy controls and for arthritis patients, respectively. Whole blood was drawn using a 21G butterfly needle into a syringe, with tourniquet applied, and anticoagulated by mixing with sodium citrate (0.32% w/v). All blood cells are from healthy controls unless indicated otherwise in figure legends. Patient data for arthritis patients are shown in Supplementary Table S1.

### 2.2 Isolation of Neutrophils and Mononuclear Cells

Whole blood was centrifuged at 130 ×*g* for 20 min and plasma was removed. For every 30 mL of whole blood, erythrocytes were depleted by sequentially layering 8 mL of 6% w/v dextran (high molecular weight, 31392-250G, Sigma-Aldrich, Poole, UK, in PBS) onto each 10 mL blood. After 15 min, the leukocyte-rich fraction was layered over Histopaque 1077 (10771, Sigma-Aldrich) and centrifuged for 30 min 450 ×*g* at room temperature to separate granulocytes from peripheral blood mononuclear cells (PBMC). Neutrophils were washed once by centrifuging at 300 ×*g* and re-suspended in phenol red-free RPMI for further use.

### 2.3 Generation of Monocyte-Derived Macrophages

A 500 μL aliquot of PBMC containing 0.9 × 10^6^ cells was seeded per well of a 24-well suspension culture plate. After 1 h incubation at 37 °C, cells were washed to remove lymphocytes and incubated with 50 ng/mL macrophage-colony stimulating factor (M-CSF; 300–25, PeproTech, London, UK) in RPMI + 10% v/v foetal bovine serum, with medium replaced on day 5. On day 7, macrophages were washed twice with PBS and used as needed.

### 2.4 Stimulation of Macrophages

Monocyte-derived macrophages were stimulated for 24 h at 37 °C with 10 ng/mL LPS (*E.Coli* 0111:B4, L2630, Sigma-Aldrich) and 20 ng/mL IFN-γ (300-02, PeproTech) or 50 ng/mL IL-4 (200-04, PeproTech). In some cases, specific inhibitors and blockers were used including 10 nM UNC-569 (445835-10MG, Millipore, Billerica, USA), 10 μg/mL anti-anxA1 (clone 1B; produced in house) or 10 μg/mL isotype control (14-4714-85, eBioscience, San Diego, USA). Neutrophil MVs were also added at the indicated concentrations. Supernatants were collected for Cytometric Bead Array for IL-12p70, IL-1β, IL-10 and TGF-β (558264, BD Biosciences, San Jose, USA) following manufacturer's instructions. Cells were detached, blocked in 160 μg/mL human IgG (G4386, Sigma-Aldrich) at 4 °C for 15 min, and labelled with 1.25 μg/mL anti-HLA_DR/DP/DQ_-FITC, 1 μg/mL anti-CD86-PE, and 4 μg/mL anti-CD206 antibodies at 4 °C for 30 min. Cells were acquired on a LSRFortessa cytometer.

### 2.5 Generation and Isolation of Neutrophil MVs

Neutrophils (2 × 10^7^ cell/mL) were stimulated with 50 ng/mL TNF-α (T0157-10UG, Sigma-Aldrich) for 20 min at 37 °C before placing on ice. Cell suspensions were centrifuged at 4,400 ×*g* at 4 °C for 15 min to pellet cells and contaminating platelets, followed by a second centrifugation at 13,000 × *g* at 4 °C for 2 min to remove residual contaminants (*e.g.* apoptotic bodies). MVs were enriched from exosomes by centrifuging at 20,000 ×*g* at 4 °C for 30 min. Exosomes were pelleted by centrifuging the supernatant at 100,000 ×*g* at 4 °C for 1 h. For both fractions, the supernatant was removed and the pellets were re-suspended in sterile PBS.

### 2.6 Nanoparticle Tracking Analysis (NTA)

MV preparations were analysed using an NS300 Nanoparticle Tracker with 488 nm scatter laser and high sensitivity camera (Malvern Instruments Ltd., Malvern, UK). For each sample, particle scatter was recorded 3 times for 60 s each under flow conditions (arbitrary speed 50) at camera level 16 and analysis threshold 5, using the NTA 3.2 acquisition and analysis software.

### 2.7 ImageStream™ Analysis of Vesicles

MVs were analysed and counted using fluorescence triggering on an ImageStream^x^ MKII imaging cytometer as described previously (Headland et al., 2014). Briefly, vesicles were labelled with 50 μM boron-dipyrromethene (BODIPY) texas red or BODIPY maleimide fluorescein (D-6116 & B10250 respectively, Life Technologies, Carlsbad, USA) as appropriate, and were acquired on their own or after labelling with either 2 μg/mL anti-CD14-PE/Cy7 (400125, Biolegend San Diego, USA), 2 μg/mL anti-CD66b-FITC (400107, Biolegend), 10 μg/mL anti-anxA1 followed by 2 μg/mL anti-mouse IgG-BV241 (405317, Biolegend) (each antibody incubation performed at 4 °C for 30 min) or with annexin A5 (anxA5) following manufacturer's instructions (51-46121E, BD Biosciences). AnxA5 positive events were gated using a sample of vesicles and anxA5 in Ca^2+^-free buffer; all protein antigen-positive events were gated using fluorescence minus one (FMO) controls.

### 2.8 MV Uptake

MVs were labelled with 5 μM CFSE before pelleting and re-suspending in PBS. In three different experiments, microvesicles (5 × 10^6^) were incubated for 15 min at room temperature with either 50 μg/mL annexin A5 (or vehicle) or 10 μg/mL anti-anxA1 (clone 1B) antibody (or isotype-matched control), or macrophages were incubated for 15 min at 37 °C with 10 nM UNC-569 or vehicle, prior to cultures in 6-well suspension wells and incubated at 37 °C for 5–90 min. Then, macrophages were detached and acquired on an ImageStream^x^ MKII.

### 2.9 Macrophage-FLS Co-Cultures

Monocyte-derived macrophages were obtained as above on 6-well 3 μm pore Transwell™ inserts (353,091, Scientific Laboratory Supplies, Nottingham, UK). Following 24 h treatment as indicated, Transwells™ were washed twice with PBS and placed into a 6-well plate containing glass coverslips with confluent FLS (408RAK-05a, Cell Applications Inc., San Diego, USA), such that the Transwell™ membrane was in contact with both fibroblasts and macrophages. After 24 h at 37 °C, 1× Golgi block (4980–03, eBioscience) was added to the culture medium for the last 6 h of culture. Fibroblasts were labelled with 0.5 μg/mL anti-VCAM-1-BV711 (744,312, BD Biosciences) and 1 μg/mL anti-CD55-APC (311311, Biolegend) antibodies at 4 °C for 30 min, fixed and permeabilised with an Intracellular Fixation & Permeabilization kit (88-8824-00, eBioscience), and labelled at 4 °C for an additional 30 min with 1 μg/mL anti-TNF-α-BV605 (502935, Biolegend), 1 μg/mL anti-IL-6-BV421 (563,279, BD Biosciences) and 1 μg/mL anti-MCP-1-PE (505903, Biolegend) antibodies. Cells were acquired on an LSRFortessa cytometer. In some cases, FLS fixed, permeabilised and labelled as above, were analysed for immunofluorescence microscopy; intracellular staining was performed with either 10 μg/mL anti-MCP-1 (MA5-17040), 15 μg/mL anti-TNF-α (MA5-23720) or 5 μg/mL anti-IL-6 (MA1-22531) antibodies. Secondary labelling was performed with anti-mouse IgG-AF488 (F-11021) and anti-rat IgG-AF488 (A-21208) as appropriate. Primary and secondary cell antibodies for immunofluorescence were purchased from ThermoFisher, Waltham, USA.

### 2.10 K/BxN Serum Induced Arthritis

All animal experiments were approved and performed under the guidelines of the Ethical Committee for the Use of Animals, Barts and The London School of Medicine and in accordance with the UK Home Office regulations (Guidance on the Operation of Animals, Scientific Procedures Act, 1986). K/BxN arthritis was induced by injecting 8-week-old male C57BL/6 mice (Charles River, UK) intraperitoneally with 100 μL of K/BxN serum on Day 0 and Day 2 as described (Patel et al., 2012). On day 3 mice were anesthetised, randomized and injected intra-articularly into the ankle synovial space with 10 μL of either 3 × 10^6^ MVs (pooled from equal numbers of vesicles from 3 human donors) in the left or right ankle, and vehicle alone in the other. Which ankle received which treatment was blinded until conclusion of the analysis. On day 5, animals were sacrificed, the femurs were removed, the ankle synovium was cut and synovia were digested in RPMI with 0.5 μg/mL collagenase D (11088866001, Sigma-Aldrich) and 40 μg/mL DNAse I (10,104,159,001, Sigma-Aldrich) under agitation at 37 °C for 30 min before collecting the cell suspension through a 70-μm cell strainer. After centrifugation at 300 × *g* for 10 min at 4 °C, cells were labelled with Zombie Aqua fixable viability dye (423101, Biolegend) to exclude dead cells followed by Fc receptor blocking, and staining with 0.125 μg/mL anti-CD11b-BV785 (101243, Biolegend), 2 μg/mL anti-F4/80-BV650 (123149, Biolegend), 2 μg/mL anti-CD86-BV421 (105031, Biolegend), 5 μg/mL anti-MHCII-AF700 (107622, Biolegend), 2 μg/mL anti-CD206-APC (141708, Biolegend) antibodies. Cells were acquired on an LSRFortessa cytometer.

### 2.11 Zymosan-Induced Peritonitis

Twelve-week old male C57BL/6 mice (Charles River, UK) were injected intraperitoneally with 1 mg zymosan (Z4250-1G, Sigma-Aldrich). Additional treatments, including 200 μg of the antagonist WRW_4_ (2262, Tocris, Bristol, UK) were injected intraperitoneally 48 h later. After a further 24 h, mice were sacrificed and the peritoneal cavity lavaged with ice-cold PBS + 2 mM EDTA. Supernatants were processed for a TGF-β mouse ELISA kit (EMTGFBI, ThermoFisher).

### 2.12 Statistical Analysis

All statistical analyses and graphing were performed in R 3.4.1 or IDEAS 6.2 for ImageStream plots. Individual biological replicates are shown for all data with bars at means; summary statistics quoted in text are mean ± standard deviation. Analyses used are indicated in each figure legend. Linear discriminant analysis (LDA) used scaled and centered variables as predictors.

## 3 Results

### 3.1 Generation and Characterization of Neutrophil MVs

**Fig. 1:**
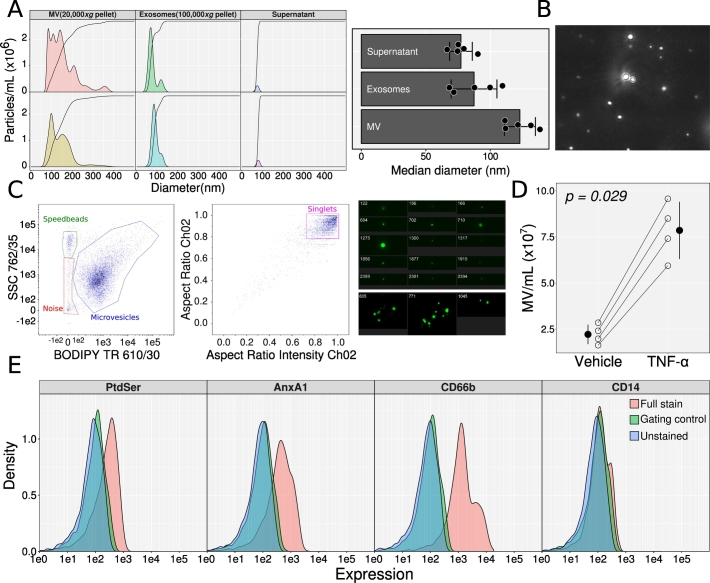
Characterization of TNF-α-induced neutrophil MV preparations. A, B) Nanosight tracking analyses (NTA) of neutrophils MVs. A: two representative density plots of MV, exosome, and supernatant after pelleting exosomes at 100,00 *×g* diameters shown with cumulative density lines overlaid. Bar plots reports the median diameter of each preparations from 5 biological replicates (mean ± standard deviation). B: representative image of particle light scatter from a sample as analysed by NTA. C–E) ImageStream analyses of neutrophil MVs. C: MVs were labelled with BODIPY texas red and acquired on an ImageStream. Gating strategy shown for gating singlet MV along with representative images of singlet and swarm vesicle events. D: Production of MVs from human neutrophils (2 × 10^7^) stimulated with 50 ng/mL TNF-α or vehicle, for 20 min at 37 °C. MVs were quantified by ImageStream. Lines connect samples from the same donor; black circles and ranges indicate mean ± standard deviation. Analysed with Wilcoxon signed rank test. E: Staining of TNF-α-induced neutrophil MVs to quantify expression of phosphatidylserine (PtdSer), annexin A1 (AnxA1), CD66b and CD14. Gating controls for CD66b and CD14 were fluorescence minus one, for AnxA1 a secondary antibody only control, and for PtdSer annexin A5 in the absence of Ca^2+^. Histograms representative of three different preparations of MVs.

NTA analysis demonstrated that MVs ranged between 70 nm and 400 nm in diameter, with median diameters of 123 ± 12 nm as assessed with 5 preparations (Fig. 1A and B). Vesicles in the 100,000 *×g* fraction and those remaining in the supernatant had median diameters of 88 ± 18 nm and 79 ± 9 nm, respectively. MV preparations labelled with BODIPY form a population of uniformly circular events that separate from noise and can be gated to exclude non-singlet events and debris (Fig. 1C) by ImageStream. Neutrophils from 4 donors stimulated with TNF-α produced 7.9 ± 1.6 × 10^7^ vesicles/mL, compared with 2.2 ± 0.5 × 10^7^ quantified in unstimulated cells (Fig. 1D). ImageStream analysis of 4 distinct preparations showed that 16% ± 7% of TNF-α-stimulated MVs expressed phosphatidylserine (*i.e.* stained with anxA5); 20% ± 6% MVs were anxA1 positive; 90% ± 4% were stained for CD66b while 0.12% ± 0.04% were CD14 positive (representative histograms in Fig. 1E), confirming, in essence, the lack of contaminating monocyte MVs.

### 3.2 Neutrophil MVs Impact on Macrophage Polarization

**Fig. 2:**
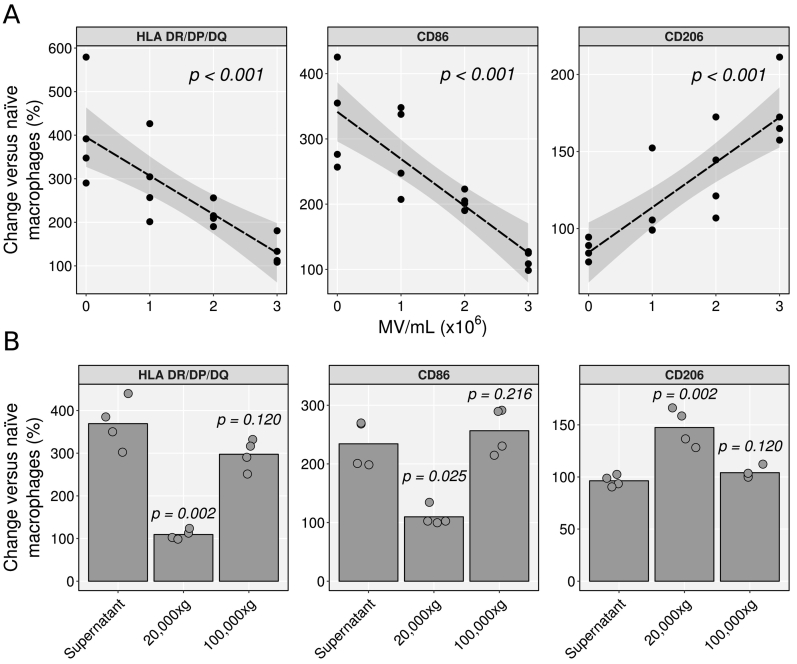
Neutrophil MVs, but not exosomes, prevent classical activation of human macrophages. A) Human monocyte-derived macrophages were treated for 24 h with 10 ng/mL LPS and 20 ng/mL IFN-γ alone or with increasing concentrations of neutrophil MVs (pooled from equal numbers of vesicles from 3 donors). Macrophage phenotype was determined by flow cytometry and compared to naïve macrophages (taken as 100%). Data analysed with a linear regression model per antigen, where least squares lines and 95% confidence bands are shown. B) Macrophages activated as above were treated with 20,000 ×*g* pellets (microvesicle-enriched), 100,000 ×*g* pellets (exosome-enriched), or residual supernatant, pooled from 2 × 10^7^ neutrophils from each of 3 donors. Macrophages were analysed by flow cytometry and compared to naïve macrophages (taken as 100%). Bars indicate group means. Data are analysed with Kruskal-Wallis test with Dunn's *post-hoc* test per antigen, where *p* values refer to the comparison to the supernatant control.

Addition of increasing concentrations of neutrophil MVs to macrophages during LPS and IFN-γ-stimulation led to a concentration-dependent decrease in HLA-DR/DP/DQ and CD86 expression, returning levels to those of unstimulated cells at 3 × 10^6^ MV/mL (Fig. 2A). A concentration-dependent increase in CD206 expression was also observed. While the MVs prevented classical activation of macrophages, they showed no effect on IL-4-mediated alternative activation (Supplementary Fig. S1). To confirm that these actions were attributable to MVs and not exosomes, sequential centrifugations of supernatants at 20,000 × *g* and then 100,000 × *g* yielded enriched preparations of MVs and exosomes, respectively. Macrophages undergoing classical activation were concomitantly treated with the MV-enriched pellet, exosome-enriched pellet, or vesicle-depleted remaining supernatant. While treatment of macrophages with the 20,000 × *g* pellet restricted upregulation of HLA-DR/DP/DQ and CD86 expression during classical activation, and increased CD206 expression, treatment with either the vesicle-free supernatant or the exosome fraction was inactive (Fig. 2B).

### 3.3 Identification of Specific MV Determinants

**Fig. 3:**
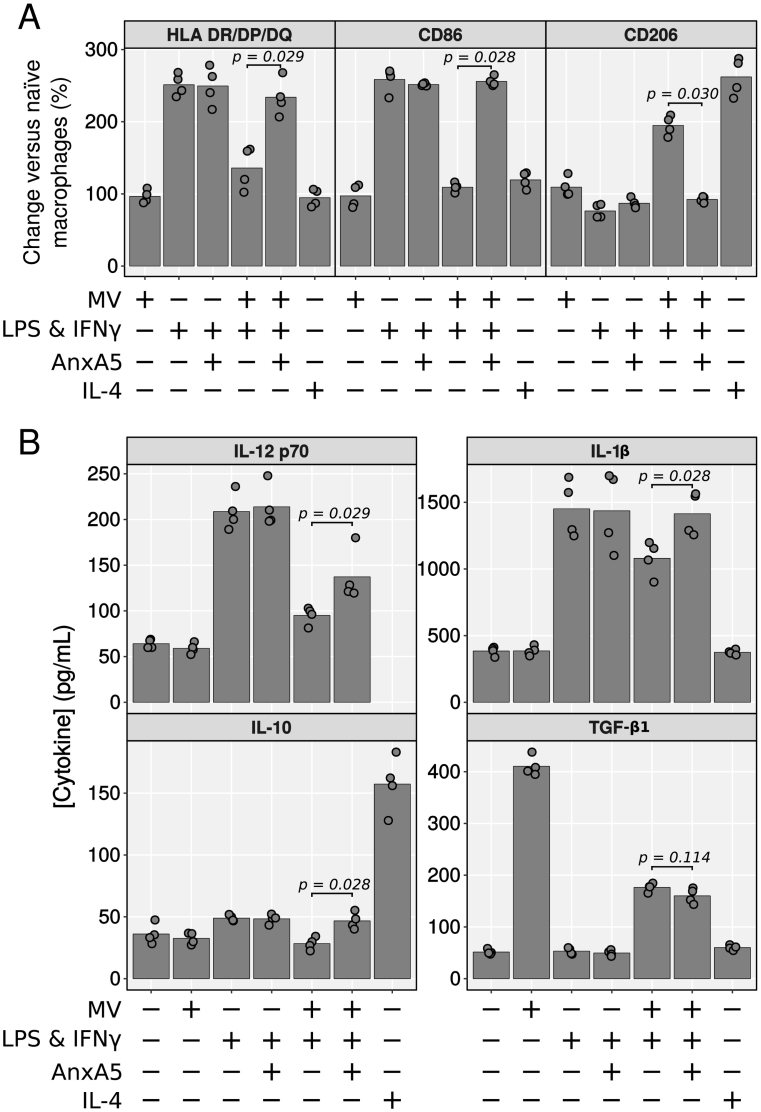
Macrophage polarization is modulated *via* phosphatidylserine. Human monocyte-derived macrophages were treated with combinations of 3 × 10^6^ /mL neutrophil MVs (pooled from 3 donors, with or without anxA5) and 10 ng/mL LPS plus 20 ng/mL IFN-γ or vehicle for 24 h. Naïve macrophages were used as negative controls; some macrophages were incubated with 50 ng/mL IL-4 for 24 h. A) Phenotypic characterization of macrophages by flow cytometry. B) Cytokines were quantified in cell supernatant using a Cytometric Bead Array. Data are median fluorescence as a percentage of untreated cells for surface proteins and absolute concentration for cytokines. Individual biological replicates are shown with bars at group means. IL-12p70 could not be detected following IL-4 stimulation. Data analysed with separate Kruskal-Wallis test with Dunn's *post-hoc* tests for each antigen.

To establish the potential functional involvement of phosphatidylserine, an “eat me” signal which promotes alternative activation of macrophages (Scott et al., 2001), exposed on the MVs, assays of classical activation of the macrophages were repeated in the presence or absence of anxA5, which binds to and buffers phosphatidylserine's actions (Fig. 3). While MVs attenuated LPS plus IFN-γ-induced expression of HLA DR/DP/DQ, CD86 (Fig. 3A), IL-12p70, IL-1β and IL-10 (Fig. 3B), and increased CD206 expression (Fig. 3A), these effects were significantly lost in MVs treated with anxA5. Soluble anxA5 was inactive on its own (Fig. 3A,B). The only mediator modulated by MV alone was TGF-β (Fig. 3B), whose production was insensitive to anxA5 addition.

**Fig. 4:**
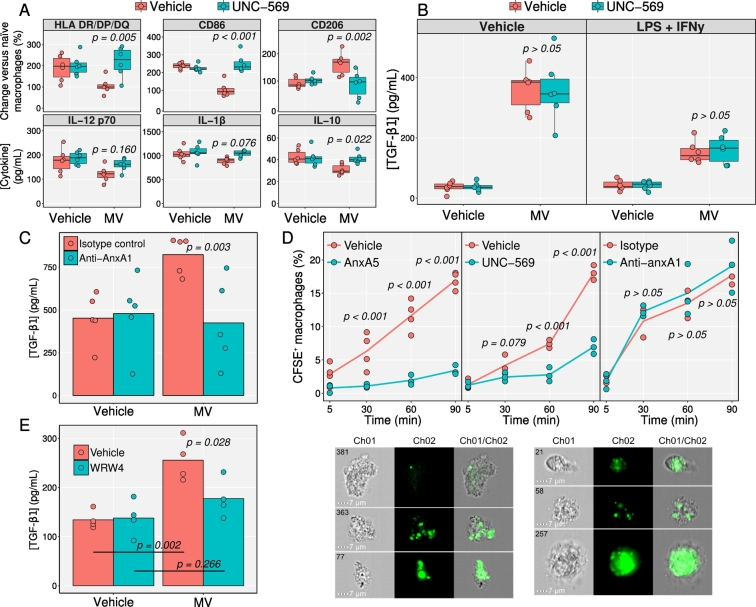
Phosphatidylserine-dependent actions of MVs are mediated by MerTK. A, B) Human monocyte-derived macrophages were treated with 3 × 10^6^ /mL neutrophil EVs (pooled from 3 donors) and 10 ng/mL LPS plus 20 ng/mL IFN-γ or vehicle for 24 h. In some cases, UNC-569 (10 nM, MerTK inhibitor) or vehicle were also added. Analyses for macrophage markers and cytokines were conducted as in Fig. 3. C) Macrophages were stimulated with 3 × 10^6^/mL MVs or vehicle, in the presence of 10 μg/mL anti-anxA1 or isotype control antibodies for 24 h before quantification of TGF-β released. D) MVs (pooled from 6 donors) were stained with 5 μM CFSE, washed, and seeded at 5 × 10^6^/mL onto macrophages. In separate experiments, MVs were either pre-treated with 50 μg/mL anxA5 (or vehicle) or 10 μg/mL anti-anxA1 (or isotype control) antibody, or macrophages were treated with 10 nM UNC-569 (or vehicle). Cells were acquired on an ImageStream after different incubation times to quantify vesicle internalisation. Representative images of macrophages containing CFSE^+^ microvesicles are shown. E) TGF-β concentration in the peritoneal lavage of mice undergoing peritonitis. Mice were injected intraperitoneally with 2 × 10^7^ MVs (pooled from 6 human donors) or vehicle alone, with or without 200 μg WRW_4_ (FPR2 antagonist), 48 h after injection with zymosan. Peritoneal cavities were lavaged 24 h later. Data in A–E analysed with separate two-way (three-way for TGF-β) ANOVA with Holm-S̆idák *post-hoc* tests for each antigen. *p* values in D compare groups at each timepoint. Data are median fluorescence as a percentage of untreated cells for surface proteins and absolute concentration for cytokines. Individual biological replicates are shown.

The phagocytosis of apoptotic moieties by macrophages is mediated in large part by the receptor-tyrosine kinase MerTK, which is a member of the tyro-3/Axl/MerTK family of receptors (Zizzo et al., 2012; Scott et al., 2001). To test if MerTK was involved in phosphatidylserine-mediated effects, macrophages were classically activated in the presence of MVs or vehicle, with or without 10 nM UNC-569 (a selective small molecule inhibitor of MerTK autophosphorylation) in a 2 × 2 factorial design (Fig. 4A,B). Protection against classical upregulation of HLA-DR/DP/DQ, CD86 and IL-10 by neutrophil MVs was lost in cells treated UNC-569, as was the upregulation of CD206 (Fig. 4A). There was a similar, though not significant, loss of protection against classical upregulation of IL-12p70 and IL-1β with UNC-569. Conversely, TGF-β secretion was independent of UNC-569 treatment (Fig. 4B).

As we identified MV anxA1 as one of the determinants for the induction of TGF-β secretion from chondrocytes (Headland et al., 2015), macrophages were treated with MVs or vehicle, in the presence of anti-anxA1 neutralizing antibody: quantification of TGF-β concentration in the supernatants showed marked TGF-β release above control in all MV-treated samples in presence of isotype control, but not in samples where endogenous anxA1 was neutralised (Fig. 4C).

To assess whether the functional engagement of phosphatidylserine or anxA1-mediated could be indirect, hence secondary to impacting on the uptake of MV by macrophage, CFSE-labelled MV were stained with anxA5 or anti-anxA1 antibody and added to macrophages, in some cases the latter being treated with UNC-569. Fig. 4D shows that while blocking the interaction between phosphatidylserine and MerTK (with anxA5 or UNC-569 respectively) significantly reduces vesicle uptake by macrophages, blockade of anxA1 did not produce any alteration. Representative images of vesicle-laden macrophages are also shown (Fig. 4D).

To confirm that anxA1-mediated induction of TGF-β was an extracellular event, likely dependent on activation of the formyl-peptide receptor type 2, mice undergoing zymosan-induced peritonitis were injected intraperitoneally with 2 × 10^7^ neutrophil MVs (pooled from 6 donors) with or without the selective FPR2 antagonist WRW_4_. Fig. 4E shows that there was a significant interaction between vesicle and WRW_4_ treatments (*p* = 0.041), where injection of MVs alone increased peritoneal TGF-β 1.9 fold over vehicle alone (*p* = 0.002), but not in the presence of WRW_4_ (*p* = 0.266; compared with WRW_4_ alone).

### 3.4 Neutrophil MVs Impact on Macrophage Downstream Functions

**Fig. 5:**
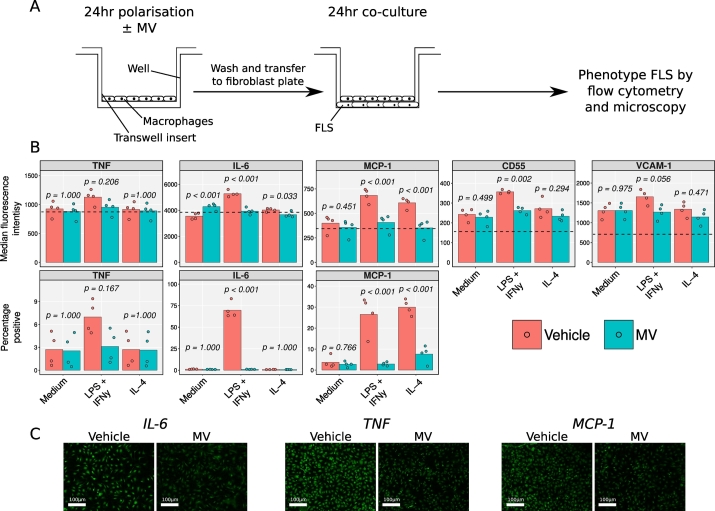
Effect of neutrophil MVs on macrophage-FLS co-culture. A) Experiment scheme. Monocyte-derived macrophages were treated with 10 ng/mL LPS and 20 ng/mL IFN-γ, 50 ng/mL IL-4 or vehicle for 24 h, in the presence of 3 × 10^6^ MV/mL or vehicle. Macrophages were washed and co-cultured with FLS for a further 24 h, after which time FLS were immunophenotyped by flow cytometry. The experiment was repeated with 3 different macrophage donors and the mean across the donors was taken for each replicate. B) Antigen expression data for each FLS donor (mean expression across co-culture with 3 different macrophage donors) where dotted lines indicate median fluorescence of isotype-matched control samples. C) Confirmatory immunofluorescence images of IL-6, TNF-α and MCP-1 expression after co-culture with macrophages after classical activation, in the presence of 3 × 10^6^ MV/mL or vehicle. Data in B analysed with separate two-way ANOVA with Holm-S̆idák *post-hoc* tests for each antigen.

Next, we queried if macrophages exposed to neutrophil MVs could interact differently with other cells, the choice being primary FLS in view of their contiguous presence in the RA synovia. FLS co-cultured with classically-activated macrophages increased their expression of TNF-α, IL-6, MCP-1, CD55 and VCAM-1, compared to those co-cultured with naïve macrophages (Fig. 5). However, macrophages treated with MVs during their activation with LPS plus IFN-γ did not induce upregulation of these antigens in the adjacent FLS.

FLS co-cultured with classically-activated macrophages expressed higher levels of all antigens measured, compared to those co-cultured with naïve macrophages, and FLS co-cultured with alternatively activated macrophages expressed higher levels of MCP-1 (Fig. 5B). These increases in expression were lost in FLS co-cultured with macrophages stimulated in the presence of MV. Modulation of FLS cytokines through macrophages ‘instructed’ by neutrophil MVs was also visualised by immunofluorescence: Fig. 5C presents characteristic images for FLS immune-reactivity of IL-6, TNF-α and MCP-1 following incubation with activated macrophages that had been exposed to vehicle or neutrophil MVs.

### 3.5 The Effect of RA Neutrophil MVs on Macrophage Polarization

**Fig. 6:**
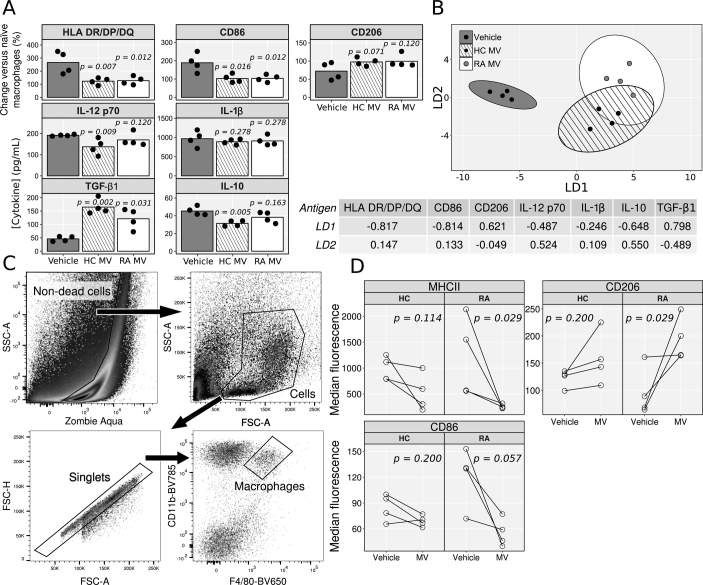
RA patient neutrophils MVs are protective *in vitro* and *in vivo*. Monocyte-derived macrophages from RA patients were stimulated with 10 ng/mL LPS and 20 ng/mL IFN-γ for 24 h, in the presence of 3 × 10^6^ MV/mL prepared and pooled from the cells of 3 healthy donors (HC), 3 RA patients (RA) or vehicle. A) Macrophage phenotype and cytokine expression after treatment. B) Results of LDA with factor loadings for each antigen. C & D) MV impact on inflammatory arthritis. On day 3 of serum induced arthritis, mice were injected intra-articularly with 3 × 10^6^ MVs from HC or RA donors (each pooled from 3 donors) into 1 ankle, and vehicle in the other. After 24 h, synovia were digested, cells extracted and macrophages phenotyped by flow cytometry (Gating strategy in C, phenotype data in D). Data in A analysed with separate Kruskal-Wallis test with Dunn's *post-hoc* tests for each antigen. Data in D analysed with separate Wilcoxon signed-rank tests for each combination of antigen and source of vesicles (HC or RA).

To test whether MVs generated from RA neutrophils displayed similar efficacy to those from healthy controls, RA patient monocyte-derived macrophages were classically activated in the presence of 3 × 10^6^ MV/mL from either healthy control or RA neutrophils, or vehicle alone (Fig. 6). MVs from healthy controls and RA patients shared similar effects across all antigens quantified (Fig. 6A). LDA showed that macrophages treated with either MV preparation separated from vehicle-treated macrophages by all antigens, most strongly HLA-DR/DP/DQ, CD86 and TGF-β, along the first discriminant factor (which accounted for 88.2% of the variability between groups; Fig. 6B and factor loadings). Macrophages treated with the two MV populations displayed similar expression profiles, separating along the second discriminant factor (which accounted for 11.8% of the variability between groups) by IL-12p70, IL-10, and TGF-β. IL-12p70 and IL-10 were more highly expressed in macrophages treated with RA MVs, whereas TGF-β (the antigen which varied the greatest between them) was more highly expressed by macrophages treated with healthy control MVs (Fig. 6B). MV_TNF_ generated from RA patient neutrophils could also out-compete the pro-inflammatory effects of total MV from RA patient synovial fluid (Supplementary Figs. S2 and S3).

### 3.6 The Effect of Neutrophil MVs in Arthritic Mice

To test whether neutrophil MVs could modulate macrophage phenotype *in vivo*, the K/BxN model of arthritis was used. Mice were randomized to receive intra-articular injection of 3 × 10^6^ MV (from healthy controls or RA neutrophils) in one ankle, and vehicle in the other. After a further 24 h, cells were immune-phenotyped by flow cytometry. Macrophages isolated from ankles which received MVs expressed lower MHCII and CD86, and higher CD206 compared to their contralateral controls. Intriguingly, these differences were more pronounced with MV_TNF_ generated from RA patient neutrophils, with joint macrophages from treated ankles exhibiting significantly lower MHCII and higher CD206 expression (Fig. 6C & D). These data supported results from a zymosan-induced peritonitis model of acute inflammation, where neutrophil MV reduced macrophage activation and increased TGF-β (Supplementary Figs. S4 and S5).

## 4 Discussion

In the burgeoning world of vesicle biology, neutrophil MVs are functionally different from other MVs as they are endowed with anti-inflammatory and pro-resolving properties. Original work of Gasser and Shifferli showed how neutrophil MVs could impact on naïve macrophages through release of TGF-β without affecting release of pro-inflammatory cytokines; however, in the presence of macrophage activators, these MVs reduced IL-8 release (Gasser and Schifferli, 2004). In our experimental settings, we confirmed and extended these observations, noting how neutrophil MVs were selective in their ability to modulate LPS + IFN-γ-induced polarization, with little effect on the M2 polarization obtained with IL-4. This is in agreement with the current literature and, we propose, might be considered in the broader pro-resolving properties of neutrophils in the context of inflammation resolution (Jones et al., 2016). Not only vesicles, but also neutrophil-derived apoptotic bodies can promote important pro-resolving properties by modifying the behaviour of surrounding cells of which the macrophage is a canonical target: macrophage efferocytosis of apoptotic neutrophils decreases expression of IL-1 and IL-6 while increasing expression of IL-10 and TGF-β (Dalli and Serhan, 2012).

A common mediator in these settings seems to be release of TGF-β. Of interest, we have recently reported the central role that this growth factor plays in the chondroprotective properties of neutrophil MVs, an effect reliant on vesicle-exposed anxA1 (Headland et al., 2015). AnxA1 also positively regulates TGF-β signalling in breast cancer cells (de Graauw et al., 2010). Modulation of TGF-β release by neutrophil MVs was observed even in non-polarised macrophages and could also be significantly augmented when cells were activated with LPS + IFN-γ. These data indicate at least a partial role for anxA1 present on the MVs as an effector for this response of the macrophage. AnxA1 did not mediate the uptake of MVs by macrophages, yet there was a genuine engagement of its receptor, formyl-peptide receptor type 2, as was indicated from the experiments conducted in the presence of its antagonist WRW_4_. It is intriguing how only a proportion of MVs expressed anxA1 on their surface, however we have also demonstrated presence of this mediator within the vesicles (Dalli et al., 2008). We could also consider how this response may be related to a general shift in macrophage phenotype towards an alternatively-activated phenotype which produces TGF-β, in line with that observed following application of IL-10 or glucocorticoids (Murray et al., 2014).

At variance from TGF-β and the anxA1/FPR2 axis, most of the markers of macrophage polarization were reliant on MV expression of phosphatidylserine, since blocking this acidic phospholipid with anxA5 prevented modulation of HLA-DR, DP & DQ and CD86 on classically-activated macrophages. We could substantiate these findings by establishing an important role for one of the phosphatidylserine receptors, the Mer tyrosine kinase or MerTK, as defined through the use of a specific inhibitor of its auto-phosphorylation and, hence, receptor activation. MerTK is the best characterised member of the tyro-3/Axl/MerTK family of receptors, especially in regards to macrophage function: MerTK activation in macrophages induces PI3 kinase-mediated phagocytosis and STAT1-mediated suppression of inflammatory cytokine signalling (Hall et al., 2003; Rothlin et al., 2007).

Taking these reported findings discussed above and the observations herein together, we would suggest that phosphatidylserine is both important for tethering, uptake, and signalling, but that other components are also at work. For example, the reduction in IL-10 production despite phosphatidylserine strongly inducing its secretion, is interesting. Moreover, while phosphatidylserine is important for internalisation, other components of the vesicles may be exerting control over phenotype, such as miRNA species.

In our experimental conditions, neutrophil MVs alone did not affect macrophage phenotype, suggesting that they inhibit classical activation rather than imparting alternative activation. A partial exception occurred in the presence of LPS and IFN-γ where the MV actually increased CD206 expression (an effect seen in neither treatment alone).

An equally important technical observation was made when MV samples were compared with exosome-enriched fractions: the data indicate that regulation of classical macrophage activation imparted by the MV preparations was attributable to the MV-enriched fraction. Equally, neutrophil-derived supernatants depleted of all microstructures were inactive. Therefore, MV uptake and specific MV effectors, including anxA1, are required to modulate macrophage polarization in our *in vitro* settings.

During RA, FLS participate in disease progression (Bottini and Firestein, 2012) by releasing cytokines and growth factors like MCP-1 (Kumkumian et al., 1989), VEGF (Palmer et al., 2008), IL-6 (Guerne et al., 1989) and GM-CSF (Parsonage et al., 2008), supporting leucocyte recruitment, delaying neutrophil apoptosis and propagating pannus hyperplasia. As the synovial lining is comprised of both fibroblasts and macrophages, it was important to explore whether neutrophil-derived MVs could impact on the macrophage-FLS crosstalk. We observed that classically-activated macrophages were instructed by the MVs to avoid maximal activation of the adjacent FLS, likely an effect downstream of the reduction of classical activation in the first place. Nevertheless, the data are suggestive of a potential novel mechanism operative in the RA synovia, where neutrophil MV-induced regulation of macrophages may have functional consequences within the inflamed microenvironment, all signalling towards an anti-inflammatory outcome.

It was important to ascertain if RA neutrophils could produce MVs able to provoke anti-inflammatory effects. Indeed, this was the case, and a combination of *in vitro* and *in vivo* analyses demonstrated a substantial overlap of actions between MVs prepared from neutrophils harvested from healthy control or RA patients. The importance of this finding lies in the potential of developing innovative therapeutic approaches based on the autologous generation of MVs from patient cells.

In line with the potential therapeutic exploitation of neutrophil MVs, we deemed it important to determine if modulation of macrophage phenotype could be attained during ongoing experimental arthritis. Following injection of the vesicles into the synovial space in the ankles of arthritic mice we could quantify an effective switch of specific macrophage markers. These effects were more prominent with neutrophil MV derived from RA patients than those generated from healthy controls, for reasons we are yet to decipher. Nevertheless, these effects on macrophage phenotype confirm the data observed *in vitro*. Future *in vivo* experiments would help define the therapeutic impact of these vesicles during on-going arthritis.

**Fig. 7:**
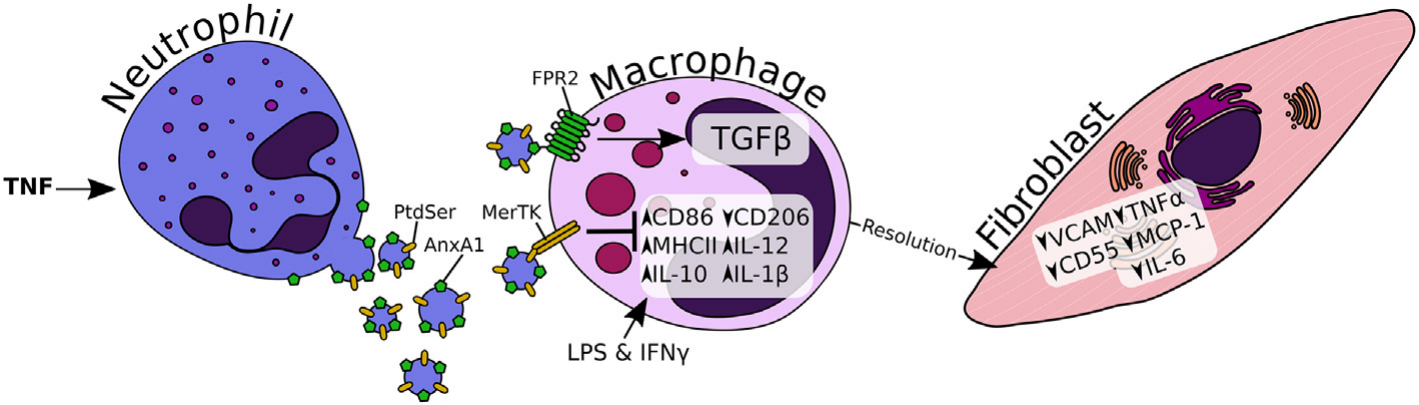
Graphical summary of hypothesis. The data presented here support the hypothesis that upon activation with tumour necrosis factor (TNF) (and potentially other inflammatory mediators), neutrophils rapidly release microvesicles into their local environment. Subsets of these vesicles express the pro-resolving protein annexin A1 (AnxA1) and/or phosphatidylserine (PtdSer) exposed on their outer membrane leaflet. The vesicles attenuate macrophage activation in response to lipopolysaccharide (LPS) and interferon gamma (IFN-γ), and influence their ability to activate other cell types, such as fibroblast-like synoviocytes in the rheumatoid synovium. Two mechanisms have been identified as responsible for these effects: AnxA1 activating FPR2 and inducing the release of transforming growth factor β (TGFβ), and PtdSer activating MerTK which blocks classical activation.

In conclusion, this new study together with current literature that includes our own work with chondrocytes and cartilage (Headland et al., 2015), presents new mechanistic evidence underpinning the anti-inflammatory and anti-arthritic properties of neutrophil MVs, summarised graphically in Fig. 7. Herein, we shed light on some of the mechanisms responsible for the effects of the MV, identifying important determinant roles for phosphatidylserine and anxA1. In our view, these data support further development of neutrophil vesicles as a ‘polypharma’ therapeutic approach for joint disease.

## Conflicts of Interest

The authors report no competing interests.

## Funding

BBSRC (studentship BB/K011782/1), MRC (project MR/P026362/1), Wellcome Trust (programme 086867/Z/08/Z) and Arthritis Research UK (Career Development Fellowship 19909 to LVN).

## Author Contributions

MP, LVN and AM planned the project. MP, LVN and HIR designed, performed and analysed experiments. FD and CP provided blood from rheumatoid arthritis patients. MP and HIR wrote the manuscript, all other authors provided advice and oversight of the manuscript.

## Appendix A Supplementary Data

Supplementary material Image 1
