# Supplementary material for: Neutrophil Microvesicles from Healthy Control and Rheumatoid Arthritis Patients Prevent the Inflammatory Activation of Macrophages
Source: eBioMedicine. 2018 Feb 7;29:60–9. doi: 10.1016/j.ebiom.2018.02.003 (PMC5925578; doi:10.1016/j.ebiom.2018.02.003)
Supplement: Supplementary file 1 — Supplementary material [file mmc1.pdf]

Supplementary Table & Figures

Table S1

Gender	Age	Rheumatoid factor	Current therapy
F	75	+	Rituximab
F	58	-	Etanercept
F	64	+	Etanercept
F	51	+	Methotrexate + hydroxychloroquine

Table of rheumatoid arthritis patient data of patients whose cells were used in the study. Monocyte-derived macrophages were generated from all four patients, MV_{TNF} were generated from and pooled from the cells of patients shaded in grey.

Figure S1

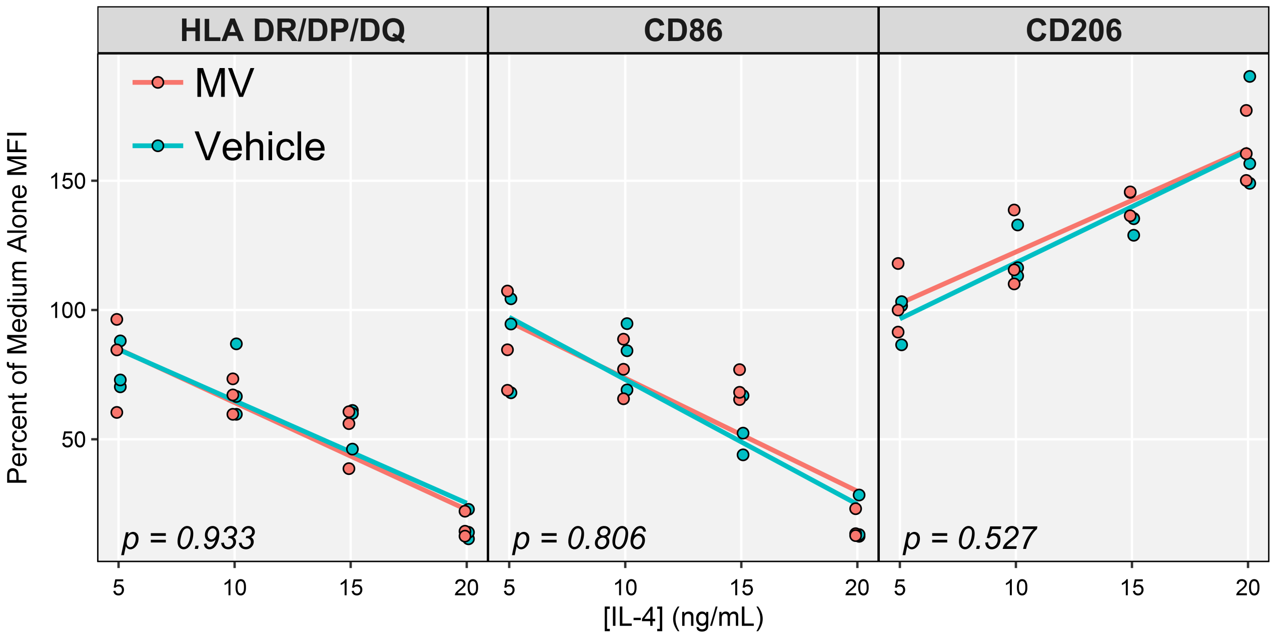

Neutrophil microvesicles do not promote M2 macrophage polarisation. Human monocyte-derived macrophages were alternatively activated with between 5ng/mL and 20ng/mL IL-4, with or without 3×10^{6} /mL MV_{TNF} (pooled between 3 donors) for 24 hours. Cells were detached, stained with antibodies against HLA-DR, DP & DQ, CD86 and CD206, and analysed using flow cytometry. Data from individual biological replicates are shown as median fluorescence as a percentage of untreated cells. Data analysed with separate ANCOVA for each antigen, where least squares lines are shown.

Figure S2

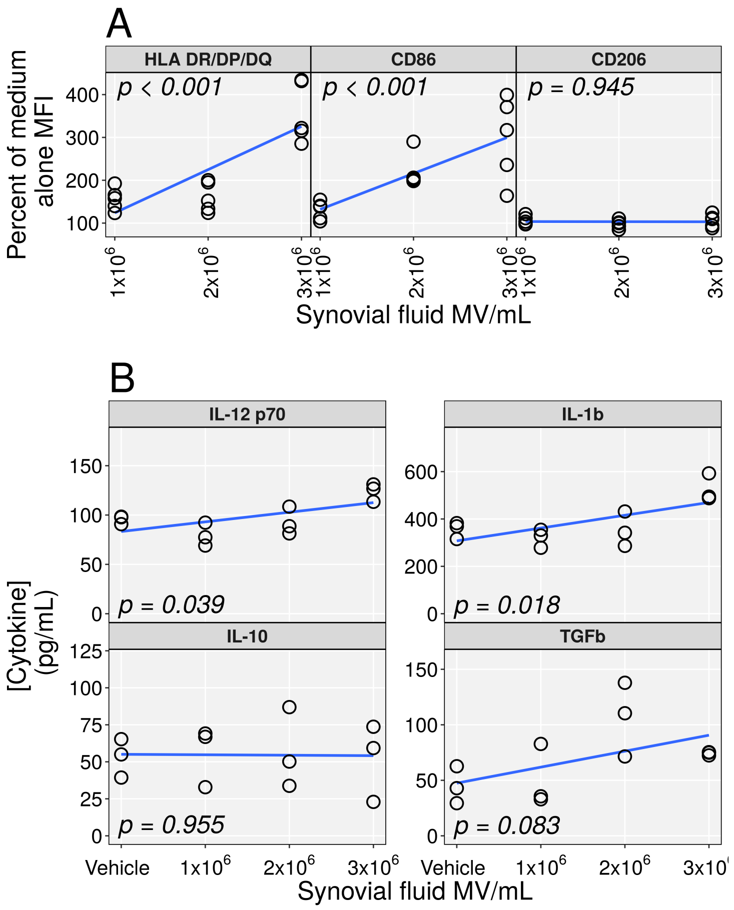

Isolated microvesicles from RA synovial fluid are pro-inflammatory. Synovial fluid from 3 rheumatoid arthritis patients (aM011-fl-RA, Cambridge Biosciences, UK) was digested with 20U/mL hyaluronidase (H3506, Sigma, USA) for 30 minutes at 37°C, depleted of cells, and centrifuged at 20,000×g to pellet microvesicles. The supernatant was aspirated (to remove soluble inflammogens), and the vesicle pellet was resuspended in PBS. Monocyte-derived macrophages were treated with medium alone, or increasing concentrations of synovial fluid microvesicles for 24hr at 37°C. Supernatants were analysed by a Cytometric Bead Array for IL-12 p70, IL-1β, IL-10 and TGFβ, and cells were detached and stained for HLA-DR, DP & DQ, CD86 and CD206 expression and analysed by flow cytometry. Data are expressed as median fluorescence as a percentage of untreated cells for surface antigens, or absolute concentration of cytokines for each donor. Data analysed with linear regression for each antigen, with least square lines.

Figure S3

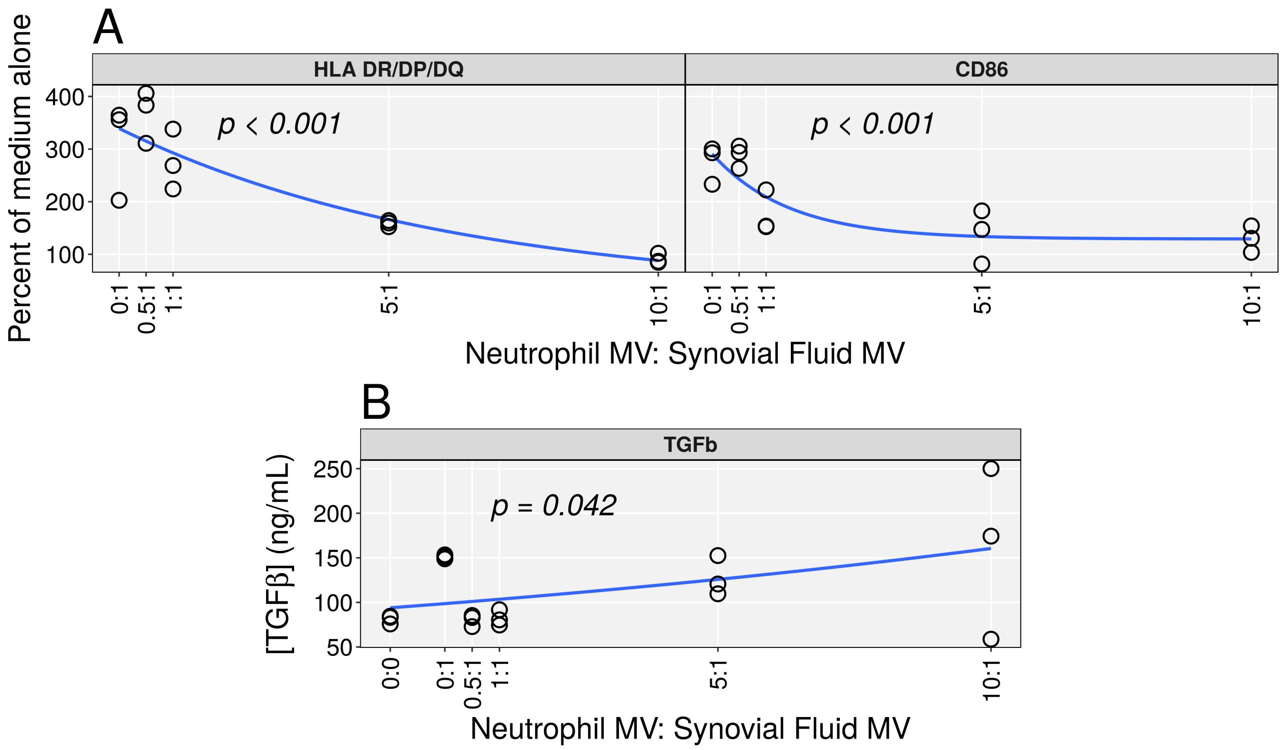

Neutrophil and synovial fluid microvesicles have opposing efficacy. Human monocyte-derived macrophages were treated for 24hr at 37°C with medium alone, or $3{\times}10^{6}$ synovial fluid microvesicles/mL (pooled between 3 rheumatoid arthritis patients) alone, or in combination with increasing ratios of neutrophil MV_{TNF}. Supernatants were collected and TGFβ concentration was measured with a Cytometric Bead Array, and cells were detached and stained with antibodies against HLA-DR, DP & DQ and CD86. Data are median fluorescence as a percentage of untreated cells for surface antigens, and absolute concentration of TGFβ for each donor. Data analysed with non-linear regression, where least squares lines are shown in blue and *p* values compare the full fits shown to intercept-only fits.

Figure S4

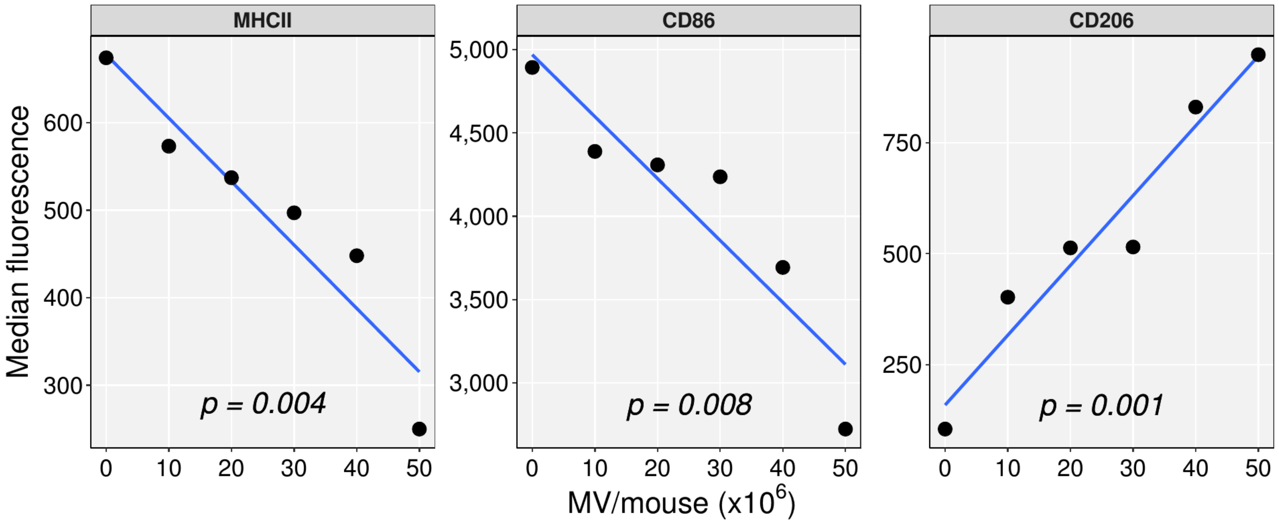

In vivo titration of MV_{TNF} on macrophage phenotype. Six, 10 week-old male C57BL/6 mice were injected with 500μL of 2mg/mL zymosan intraperitoneally. After 24hr, mice were randomised to a range of doses of human MV_{TNF} (pooled between 6 donors) injected intraperitoneally in 500μL. A further 24hr later, mice were culled and a peritoneal lavage performed with 2mM ethylenediaminetetraacetic acid (EDTA). Cells were stained with antibodies against F4/80, MHCII, CD86 and CD206, and analysed by flow cytometry. Data are median fluorescence of each antigen of F4/80$^+$ cells, for each mouse. Data analysed with linear regression for each antigen, where least square lines are shown. Both treatment and analysis were blinded.

Figure S5

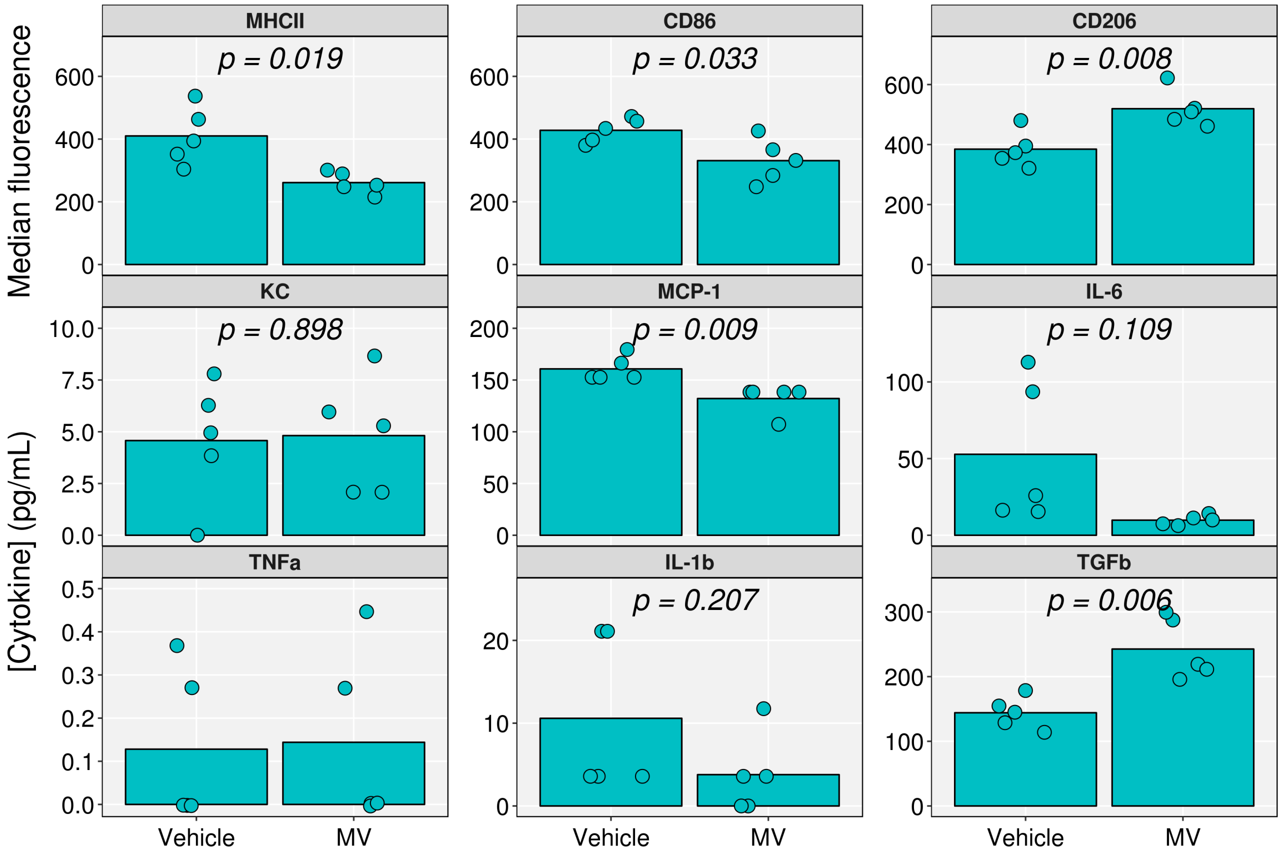

Confirmatory, two group peritonitis. Ten week-old male C57BL/6 mice were injected with 500μL of 2mg/mL zymosan intraperitoneally. After 24hr, mice were randomised to intraperitoneal injection of 2×10^7 human MV_{TNF} (pooled between 6 donors) or vehicle alone (both 500μL). A further 24hr later, mice were culled and a peritoneal lavage performed with 2mM EDTA. Cells were stained with antibodies against F4/80, MHCII, CD86 and CD206, and analysed by flow cytometry. Cell-free lavage fluid was sent to Labospace for Luminex cytokine profiling. Data are median fluorescence of each surface antigen of singlet F4/80$^+$ cells, or absolute concentration of cytokine, for each mouse. Data analysed with separate two-tailed t tests for each antigen, with bars at group means. Both treatment and analysis were blinded.
